# *In vivo* calcium imaging shows that satellite glial cells have increased activity in painful states

**DOI:** 10.1093/braincomms/fcae013

**Published:** 2024-01-18

**Authors:** Sara E Jager, George Goodwin, Kim I Chisholm, Franziska Denk

**Affiliations:** Wolfson Centre for Age-related Diseases, King’s College London, Guy’s Campus, London SE1 1UL, UK; Molecular Neuropharmacology and Genetics Laboratory, Department of Neuroscience, Faculty of Health and Medical Sciences, University of Copenhagen, 2200 Copenhagen, Denmark; Wolfson Centre for Age-related Diseases, King’s College London, Guy’s Campus, London SE1 1UL, UK; Pain Centre Versus Arthritis, School of Life Sciences, University of Nottingham, Nottingham NG5 1PB, UK; Wolfson Centre for Age-related Diseases, King’s College London, Guy’s Campus, London SE1 1UL, UK

**Keywords:** *in vivo* Ca^2+^ imaging, satellite glial cells, peripheral nerve injury, dorsal root ganglion, neuropathic pain

## Abstract

Satellite glial cells are important for proper neuronal function of primary sensory neurons for which they provide homeostatic support. Most research on satellite glial cell function has been performed with *in vitro* studies, but recent advances in calcium imaging and transgenic mouse models have enabled this first *in vivo* study of single-cell satellite glial cell function in mouse models of inflammation and neuropathic pain. We found that in naïve conditions, satellite glial cells do not respond in a time-locked fashion to neuronal firing. In painful inflammatory and neuropathic states, we detected time-locked signals in a subset of satellite glial cells, but only with suprathreshold stimulation of the sciatic nerve. Surprisingly, therefore, we conclude that most calcium signals in satellite glial cells seem to develop at arbitrary intervals not directly linked to neuronal activity patterns. More in line with expectations, our experiments also revealed that the number of active satellite glial cells was increased under conditions of inflammation or nerve injury. This could reflect the increased requirement for homeostatic support across dorsal root ganglion neuron populations, which are more active during such painful states.

## Teaser

Jager *et al*. report that satellite glial cells have increased calcium activity in painful states although the activity does not seem to be time-locked with neuronal activity as expected. Instead, the satellite glial cells seem to work according to their own schedule.

## Graphical Abstract

**Graphical Abstract:**
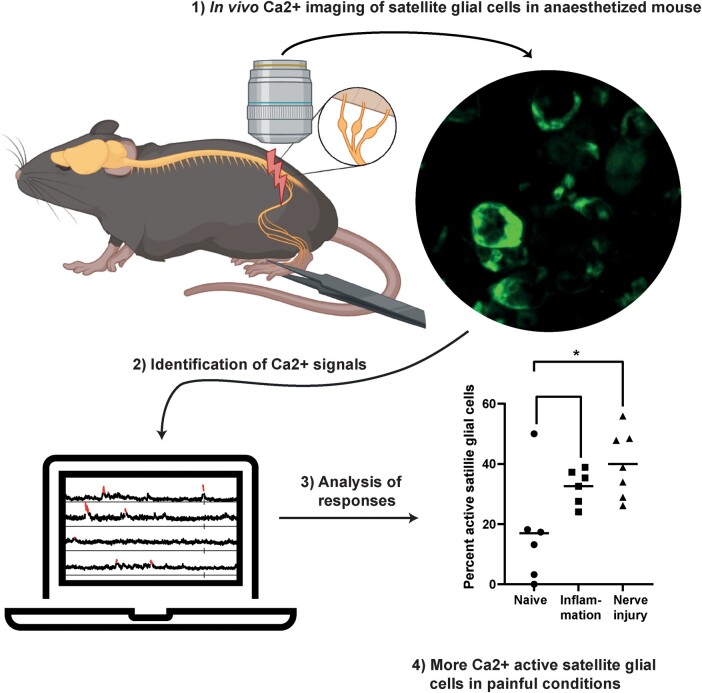

## Introduction

Our ability to feel touch, temperature or pain is reliant on primary sensory neurons, which connect our spinal cord and brain to the environment. What is often overlooked, however, is that these neurons cannot function in isolation but require homeostatic support from Schwann cells and satellite glial cells (SGCs). The cell bodies of peripheral sensory neurons are enveloped by SGCs^1,2^; in rodents, it appears that the larger the neuron, the more SGCs it has—with up to 10 reported per cell.^3^

SGCs are believed to provide buffering functions, e.g. by taking up potassium^4,5^ and neurotransmitters from the extracellular space surrounding the neurons.^6^ SGCs are thus crucial for maintaining homeostasis, as sensory neurons require tight environmental control in order to maintain their membrane potential and firing properties. There is also evidence to suggest that SGC function changes when sensory neurons are injured or affected by disease.^7^ For example, studies report that SGCs show increased expression of gap junction transcripts and proteins after traumatic nerve injury, strengthening inter-SGC connectivity.^4,8-10^ These changes could be a reflection of SGCs responding to altered neuronal function in an injured or diseased environment.

Despite their pivotal contribution to somatosensation, many crucial questions about SGC communication remain unanswered. The primary reason for this is likely that they are very difficult to study. Without antibodies, SGCs are hardly visible, which means that their presence in peripheral neuronal culture systems often goes unnoticed.^11^ On the other hand, SGCs cultured in the absence of neurons rapidly regress into a Schwann cell–like precursor phenotype,^12,13^ which means that any *in vitro* studies of pure SGCs ideally have to be undertaken within a few days of extraction. Luckily, progress in optical imaging techniques has meant that SGC function can now finally be studied *in vivo*, using calcium imaging of dorsal root ganglia (DRG),^14^ the site of peripheral neuron-SGC units.

In this study, we used a transgenic approach, using *Fabp7*, an SGC marker gene,^15^ to drive expression of the calcium sensor GCaMP6s. The transgenic mouse we chose has previously been used in the SGC field, seemingly successfully.^15,16^ Equally, the GCaMP6s imaging has been increasingly used to assess cellular activity in DRG *in vivo.*^17-20^ Here, we set out to use these techniques to investigate the following key questions:

1. Do SGCs generate calcium transients in response to peripheral neuron stimulation?
  We know from *in vitro* experiments that ATP is released by neuronal cell bodies in an activity-dependent manner.^21^ The ATP has then been shown to bind to purinergic receptors on SGCs, where it results in an intracellular calcium signal that affects downstream cellular functions, including communication with neurons.^21,22^ The resulting calcium waves can be further propagated to neighbouring SGCs or neurons via gap junctions.^23^ However, to date, all of this work has been conducted *in vitro*, and it is therefore not clear whether neuronal activity can also generate calcium signals in SGCs in a physiological, *in vivo* situation.
2. Do SGCs increase their responses when the peripheral nervous system is sensitized, e.g. by inflammation or injury?
  This has frequently been suggested based on *in vitro* studies. Thus, stimulation with ATP was shown to result in a higher calcium signal in primary SGC cultures derived from inflammatory models.^24-26^ Most of the other literature is based on indirect measures of SGC activation. For example, many have observed an increase in gap junctions between SGCs upon sensitization,^4,8-10^ which should allow for increased propagation of signals between cells. Others, including ourselves, have identified molecular changes that indicate increased SGC responses in disease states, such as alterations in mRNA expression related to cholesterol^27^ and lipid metabolism^15^ and regulation of various proteins such as rat GFAP,^28,29^ Hmgcs1^30^ and Kir4.1.^31,32^

In this paper, we conduct *in vivo* calcium imaging to obtain a direct measure of whether (i) neuronal activity can induce calcium transients in healthy SGCs and (ii) whether inflammation or neuropathy can increase this signalling mechanism.

## Materials and methods

### Transgenic animals for investigating SGCs

All mice were housed under standard conditions with 12 h light/dark cycles and free access to chow and water. To create mice with transgene expression in SGCs, the Cre-dependent mouse strains flox-STOP-*GCaMP6s* (The Jackson Laboratory, Bar Harbor, ME, USA, 028866) and the nuclear-tagged reporter line flox-STOP-*Sun1GFP* (The Jackson Laboratory, #030958) were crossed with the *Fabp7*-creER mouse strain (BRC Riken, Ibaraki, Japan, #RBRC10697).^33^ The mice were bred as homozygotes for either GCaMP6s or Sun1GFP and hemizygote for *Fabp7*-CreER. The genotypes of the mice were confirmed using standard polymerase chain reaction conditions with the primers listed in Table 1. For expression of GCaMP6s or green fluorescent protein (GFP), the mice were given 180 mg/ml tamoxifen (Merck KGaA, Darmstadt, Germany, #T5648-1G) dissolved in corn oil (Merck, #C8267-500ML) with oral gavage for 5 consecutive days. We found this to be the most effective tamoxifen administration protocol, having also tried delivering tamoxifen via food and via serial intraperitoneal injections. No protocol we tried enabled us to achieve consistent transgene expression. Two weeks were allowed between the tamoxifen treatment and further procedures such as nerve ligation and *in vivo* Ca^2+^ imaging. All mice used in the study were between 8 and 17 weeks of age. All animal experiments were performed in accordance with the UK Home Office Legislation (Scientific Procedures Act, 1986) and were approved by the Home Office to be conducted at King’s College London under project licence number P57A189DF.

**Table 1:** Primers used for genotyping

Genotype	Primer name	Primer sequence
GCaMP6	GCAMP6_wt_rev	CAGGACAACGCCCACACA
	GCAMP6_wt_fw	AAGGGAGCTGCAGTGGAGTA
	GCAMP6_mut_fw	GAAGGGCATCGACTTCAAGG
	GCAMP6_mut_rev	TCTGCGATCTGCTCTTCAGT
Sun1GFP	Sun1GFP_wt_fw	GCACTTGCTCTCCCAAAGTC
	Sun1GFP_wt_rw	CATAGTCTAACTCGCGACACTG
	Sun1GFP_mutGFP_rev	CTGAACTTGTGGCCGTTTAC
	Sun1GFP_mutSun1_fw	ACACTTGCCTCTACCGGTTC
Fabp7creER	Fabp7CreER_common_fw	TTGACCTACTCCGCTAACCC
	Fabp7CreER_mut_rev	CTTGCGAACCTCATCACTCGT
	Fabp7CreER_wt_rev	CGCAAGAAGGCAGGAAAATACC

Annealing temperature at 59°C for all reactions.

### Immunohistochemistry of DRGs

DRGs from tamoxifen-treated *Fabp7*-CreER-*GCaMP6s* mice were dissected following transcardial perfusion with phosphate buffered saline (PBS) and 4% paraformaldehyde (PFA). The DRGs were post-fixed for 2 h in 4% PFA before they were washed in PBS and incubated overnight in 30% sucrose for cryoprotection. The tissue was embedded in OCT (CellPath Ltd, Newton, UK, #KMA-0100-00A) and stored at −80°C. The embedded tissue was cut at 10 µm on a cryostat and collected on Superfrost plus slides (ThermoFisher Scientific, Waltham, MA, USA, #J1800AMNZ). The slides were baked for 30 min at 50°C to ensure adhesion of the tissue sections before the OCT was washed off with PBS. A hydrophobic barrier was drawn (Vector Laboratories, Newark, CA, USA, #H-4000) around the tissue sections. When the barrier was dry, the slides were washed in PBS with 0.3% triton x-100 (Santa Cruz Biotechnology Inc., Dallas, TX, USA, #sc-29112A), and the tissue sections were incubated with blocking solution (10% donkey serum and 0.3% triton x-100 in PBS) for at least 1 h in a humified chamber. Next, they were incubated in a primary antibody mix (see Table 2) diluted in a blocking solution overnight at room temperature. The following day, the tissue sections were washed for 3 × 5 min in PBS with 0.3% triton x-100 before they were incubated in a secondary antibody mix (see Table 3) diluted in blocking buffer for 4 h at room temperature protected from light. The slides were washed for 3 × 5 min in PBS with 0.3% triton x-100 before they were coverslipped using a mounting medium containing 4′,6-diamidino-2-phenylindole (DAPI) (Invitrogen, Waltham, MA, USA, #00-4959-52). The slides were imaged with a Zeiss LSM 710 confocal microscope and quantified using ImageJ. For any given experiment, four to six sections were stained from each mouse. The stained cells were quantified and reported as absolute counts or percentages of the total amount of cells as determined based on the number of nuclei. For each staining, tissue from six to seven mice was used, enough to detect any sizable recombination events outside of our target cell type. For example, a one-sample *t*-test with *n* = 6 would give an 80% chance to detect significant recombination in a cell type other than SGCs (*d* = 1.79, i.e. a change from 0 to 4% recombination with a standard deviation of the mean difference as large as 5).

**Table 2:** Primary antibodies used for IHC, DRG whole mount or flow cytometry (flow)

Application	Antibody	Dilution	Conjugation	Catalogue #	Supplier
IHC	Rabbit α-GS	1:5000	No	Ab49873	Abcam
IHC	Chicken α-MPZ	1:250	No	Ab39375	Abcam
IHC	Rabbit α-GFP	1:250	No	A11122	Invitrogen
IHC	Goat α-Fabp7	1:500	No	AF3166	R&D
IHC/whole mount	Chicken α-GFP	1:5000	No	Ab13970	Abcam
Whole mount	Rabbit α-ATF3	1:500	No	NBP1-85816	Novus
Flow	α-CD31	1:300	BV711	740680	BD Biosciences
Flow	α-CD11b	1:50	PE	101208	Biolegend
Flow	α-CD45	1:300	PerCP Cy5.5	45-0451-80	eBiosciences
Flow	Rabbit α-GS	1:200	APC	Ab49873, Ab201807	Abcam

The last antibody in the table was conjugated in-house with APC. The catalogue number for the conjugation kit is added after the catalogue number for the antibody in the table. APC, allophycocyanin; IHC, immunohistochemistry.

**Table 3:** Secondary antibodies used for IHC or DRG whole mount

Application	Antibody	Dilution	Fluorophore	Catalogue #	Supplier
IHC	Donkey α-rabbit	1:250	Dylight 650	Ab96894	Abcam
IHC	Donkey α-chicken	1:1000	Alexa Fluor 488	SAB4600031-50UL	Sigma-Aldrich
IHC	Donkey α-goat	1:1000	Alexa Fluor 546	A11057	Invitrogen
Whole mount	Donkey α-rabbit	1:1000	Alexa Fluor 568	A10042	Invitrogen
Whole mount	Goat α-chicken	1:1000	Alexa Fluor 488	A11039	Invitrogen

IHC, immunohistochemistry.

### Flow cytometry of DRGs

L3–L5 DRGs from tamoxifen-treated *Fabp7*-CreER-*Sun1GFP* mice were dissected following transcardial perfusion with PBS.^34^ The DRGs were enzymatically dissociated by incubation with a digestion mix containing F12 with 3 mg/ml dispase II (Sigma Aldrich, #04942078001), 12.5 mg/ml collagenase Type IA (Sigma Aldrich, #C9891) and 10 mg/ml DNase I (Sigma Aldrich #10104159001) for 45 min at 37°C. The cells were triturated with a p1000 pipette tip until homogenous and filtered through a 70-µm cell strainer into an Eppendorf tube. They were spun down at 200 × *g* for 5 min and resuspended in Hank’s balanced salt solution (HBSS; Gibco, New York, NY, USA, #14175095). Next, the cells were moved to a 96-well plate and protected from light thereon. Staining proceeded with 50 µl of near-IR live/dead (Invitrogen #L34975) diluted 1:1500 in HBSS. After a 30-min incubation on ice, we added 50 µl of primary antibody mix (see Table 2) diluted in flowcytometry (FC) buffer containing Hank's balanced salt solution (HBSS) with 0.4% bovine serum albumin (Sigma-Aldrich, #A3983), 15 mM 4-(2-hydroxyethyl)-1-piperazineethanesulfonic acid (HEPES) (Gibco, #15630080) and 2 mM ethylenediaminetetraacetic acid (EDTA) (Invitrogen, #15575-038). This was left for a further 30 min on ice before cells were spun down at 200 × *g* for 5 min and fixed in 4% PFA for 10 min followed by permeabilization in perm buffer (Invitrogen, #00-8333-56) with 10 µg/ml of Hoechst (ThermoFisher Scientific, #62249) for 5 min at room temperature. To perform the intracellular staining, the cells were incubated with the glutamine synthetase (GS) antibody (see Table 2) diluted in perm buffer for 30 min on ice. Finally, the cells were spun down at 200 × *g* for 5 min and resuspended in FC buffer followed by flow cytometric analysis at the NIHR BRC flow core facility at King’s College London on a BD Fortessa or BD FACSCanto. Fluorescence minus one stainings were used as staining controls. For each flow cytometric analysis, tissue from five mice was used, which powered us to detect significant off-target recombination (80% chance to detect *d* = 2.02 with a one-sample *t*-test and alpha = 0.05, i.e. a change of 0–4.25% recombination).

### Neuropathic pain model

Partial sciatic nerve ligation (pSNL) was performed in the left hindleg using a paradigm originally developed in the rat.^35^ In short, mice were administered 0.05 mg/kg of the analgesic buprenorphine (Ceva, Libourne, France, #Vetergesic) and anaesthetized with 2% isoflurane (Henry Schein, Melville, NY, USA, #988-3245). After shaving the fur covering the left hip and leg region, a skin incision was made, and the sciatic nerve was exposed by blunt dissection of the muscle layer. Approximately, two-third of the sciatic nerve was tightly ligated with a 5.0 suture (Ethicon, Cincinnati, OH, USA, W9982). The incision was closed with a surgical clip (World Precision Instruments, Sarasota, FL, USA, #500433), before mice were placed in a clean cage and left to recover in the animal unit. The weight of the mice was monitored daily until the pre-procedure weight was re-established. Mice were used for imaging experiments on Day 7 after the procedure.

### Inflammatory pain model

Mice were anaesthetized with 2% isoflurane and injected with 20 µl of complete Freund’s adjuvant (CFA), 1 mg/ml (Sigma, #F5881-10ML) in the left hind paw. They were used the following day for *in vivo* Ca^2+^-imaging experiments.

### *In vivo* Ca^2+^ imaging

Terminal *in vivo* Ca^2+^ imaging was performed on SGCs in *Fabp7*-CreER-*GCaMP6s* mice and on neurons in wild-type mice labelled through viral transduction of GCaMP6s. For the latter, 5 µl of AAV9 pAAV-Syn-*GCaMP6s*-WPRE.SV40 virus (Addgene, Watertown, MA, USA, #100843-AAV9) was injected into C57BL/6J pups 3–6 days after birth as previously described.^36^

For imaging, anaesthesia was induced with an i.p. injection of 12.5% w/v urethane (Merck, U2500-100G), and surgical depth was achieved using ∼2% isoflurane. The exact level of isoflurane was determined based on hindlimb and corneal reflex activity. The core body temperature was maintained close to 37°C using a homeothermic heating map with a rectal probe (FHC, Bowdoin, ME, USA). An incision was made in the skin on the back, and the muscle overlying L3, L4 and L5 DRG was removed. The L4 DRG was identified based on the position of the hip bones and the vertebra. The bone covering the L4 DRG was carefully removed, and the underlying epineurium and dura mater over the DRG were washed and kept moist with saline. The exposed L4 DRG was covered with silicone elastomer (World Precision Instruments, #Kwik-sil) to avoid drying and maintain a physiological environment as described previously.^20^ The mouse was then placed under an Eclipse Ni-E FN upright confocal/multiphoton microscope (Nikon, Tokyo, Japan). All images were acquired using a 20× dry objective and a 488-nm argon ion laser line. Time series recordings were made with a resolution of 512 × 512 pixels and a fully open pinhole. Image acquisition was at either 1.8 or 3.6 Hz depending on experimental requirements. When possible, up to three distinct areas of the DRG were imaged for each mouse to record from as many SGCs as possible. At the end of the experiment, the mouse was culled with an intracardial injection of pentobarbital (Euthatal; Merial, Duluth, GA, USA, #P02601A). Data were collected from six naïve mice (two male and four female), six CFA-treated mice (one male and five female) and seven pSNL mice (two male and five female) between 8 and 17 weeks of age. We chose these sample sizes as we were looking for large effects between biological replicates, and with *n* = 6, one has an 80% chance to detect *f* = 0.89 with alpha = 0.05 using a one-way ANOVA, as we did. Of note, these considerations are at their most conservative, as we are not taking into account the large number of cells we are recording from each mouse. Due to the nature of these experiments, it was not possible for the experimenter to be blind to treatment conditions when collecting the data.

### Activation of neurons during *in vivo* Ca^2+^ imaging

To activate neurons in the L4 DRG, the sciatic nerve was electrically stimulated using a custom-made cuff electrode with insulated steel wire as previously described.^20^ Pulses of 500 µA amplitude and a duration of 400 µs were used to activate A fibres in the sciatic nerve, while suprathreshold stimulation with pulses of 1 mA amplitude and a duration of 1 ms were used to activate both A fibres and C fibres.^20^ Furthermore, neurons in the sciatic nerve were activated by squeezing the hind paw with a pair of tweezers for a duration of 5 s. We could not visualize the resulting neuronal activity in our *Fabp7*-CreER-*GCaMP6s* mice, since any neuronal GCaMP6s signal would have interfered with that in SGCs and vice versa. However, we know from our own work (Supplementary Fig. 1) and that of others^17,20,36^ that electrical stimulation of the sciatic nerve very robustly induces activation of the vast majority of neurons within a field of view during our calcium imaging setup.

### Analysis of *in vivo* Ca^2+^-imaging data

Drift in the time series recordings was corrected using the registration framework in Suite2p v0.10.3.^37^ To extract the Ca^2+^ traces, regions of interests (ROIs) corresponding to individual SGCs were identified using Suite2p together with Cellpose v1.0^38^ (Supplementary Fig. 2). The detected ROIs were quality checked manually leading to exclusion of ROIs on the edge of the video and the addition of extra ROIs when needed.

The output from Suite2p (‘Fall.mat’) was modified with a custom MATLAB script, which dynamically subtracts the background neuropil fluorescence, i.e. signal in the pixels surrounding each ROI.

Next, the time series recordings were analysed to identify the Ca^2+^ responses. The threshold for a positive response was taken as a signal above 25% of the mean fluorescent signal for the full video (or until pentobarbital administration) plus 2 × standard deviation. For 13 ROIs (4 in naïve condition, 4 in CFA condition and 5 in pSNL condition), the mean was too high to be used as a baseline because of a very high level of Ca^2+^ activity throughout the recording. In these cases, a baseline interval without activity was identified individually for each ROI. Responses only spanning one frame were determined to be noise and were thus removed from further analysis. Since our computational pipelines are largely automated, these analyses were not generated by an experimenter blind to treatment conditions.

Visualization with raster plots was generated in MATLAB using plotSpikeRaster_v1.2,^39^ after the data were changed to a binary format based on whether the fluorescent signal reached the threshold or not.

The frequency of activity was calculated for each active SGC in a mouse as the number of Ca^2+^ responses per minute. A SGC was considered active and thus included in this analysis, if it had at least one Ca^2+^ response during the imaging session.

A Ca^2+^ response was determined to be time locked if it was initiated within 7.5 s of the noted time for the stimulation of the neurons. Thus, the chance for one cell to appear time locked was calculated as *F*_B_ = *R* × 7.5 s, where *F*_B_ is the number of background firing events in every 7.5-s interval and *R* is the average firing rate of cells per second. To determine the number of time-locked responses expected by chance, we multiplied the background firing events of one cell (per 7.5-s interval) with the number of cells in a given dataset.

Correlation analysis was performed to investigate whether SGCs surrounding individual injured neurons are more correlated than SGCs surrounding individual uninjured neurons. The ROIs identifying SGCs surrounding the same neurons were identified manually, and their fluorescent traces were compared with Pearson’s correlation in MATLAB.

### Whole-mount staining of DRGs

Following *in vivo* Ca^2+^ imaging, DRGs were dissected and immersion fixed in 4% PFA for at least 3 h. They were then washed in PBS, followed by incubation in a blocking solution (10% donkey serum and 0.3% triton x-100 in PBS) for at least 1 h. Next, the DRGs were stained with a primary antibody mix (see Table 2) diluted in a blocking solution for 3 days at room temperature and protected from light. They were then washed 3 × 5 min in PBS with 0.3% triton x-100, before being incubated with a secondary antibody mix (see Table 3) diluted in a blocking solution for 2 days at room temperature and protected from light. The DRGs were imaged in two rounds to increase the chance of identifying the same SGCs visualized during *in vivo* Ca^2+^ imaging. First, we used a fluorescent microscope (Zeiss Axio Imager Z1) covered only in PBS to retain as much original 3D structure as possible. Next, the DRGs were covered with a mounting medium and a glass slide prior to imaging on a Zeiss LSM 710 confocal microscope with Z-stacks. The images were used to identify which SGCs in the *in vivo* Ca^2+^-imaging data were surrounding injured neurons in the pSNL condition. Great care was taken to avoid false negatives, i.e. a neuron was only identified as uninjured if it had been imaged in its entirety in the *z*-plane, ensuring that no ATF3+ signal in the nucleus could have been missed. Moreover, SGCs were only included in the analysis when it was clear whether they were surrounding an ATF3+ positive or negative neuron. The analysis was performed by an investigator blind to treatment conditions and fluorescent traces.

### Statistical analysis

All statistical analyses were performed in GraphPad Prism 9. For comparisons between the three groups (Naïve, CFA and pSNL), ordinary one-way ANOVA followed by Tukey’s multiple comparisons test was used. For the comparison between the percentage of SGCs surrounding ATF3-positive neurons in naïve versus pSNL animals, a two-tailed Mann–Whitney test was used. For the comparison between per cent active SGCs surrounding injured or uninjured neurons, a paired *t*-test was used. Finally, for the comparison of the distribution of the Pearson correlations, a non-parametric two-tailed Mann–Whitney test was used.

## Results

**Figure 1:**
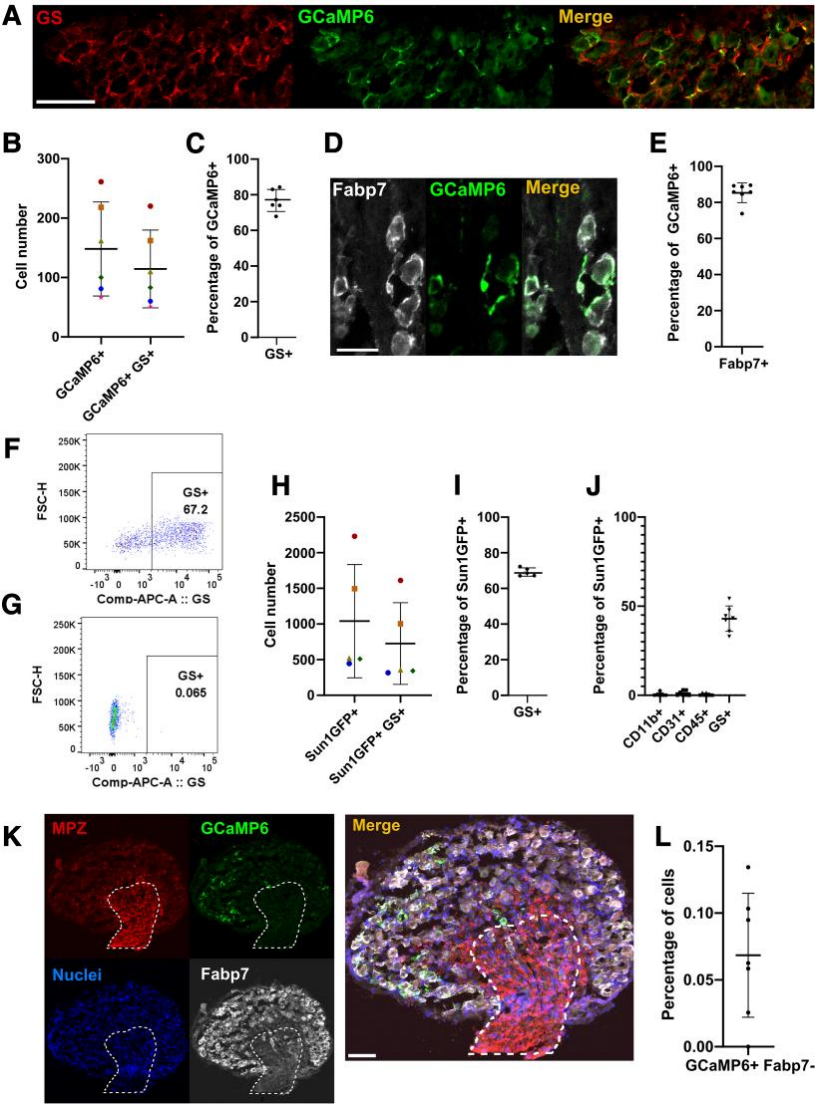
**Using *Fabp7*-CreER-*GCaMP6s* mice to target a calcium sensor to SGCs. (A)** Representative image of immunofluorescent staining of a DRG from the *Fabp7*-CreER-*GCaMP6s* line. SGCs were visualized with an antibody against the SGC marker GS, while GCaMP6s signal was enhanced with an antibody against GFP. Scale bar = 100 µm. **(B)** Quantification of immunofluorescence. Shown are the absolute counts of GCaMP6s+ positive cells and GCAMP6s+/GS+ double positive cells. Data from *n* = 6 mice (same colour = data from the same animal). Standard deviation and mean values are shown in the graph. Six sections were counted for each mouse, and the counts were added to feed into this plot. **(C)** Plot of the percentage of GCaMP6s+ cells also positive for GS+, calculated from the values in B. Error bars = standard deviation. **(D)** Representative image of immunofluorescent staining of DRG tissue from the *Fabp7*-CreER-*GCaMP6s* line. SGC was visualized with an antibody against fatty acid–binding protein 7 (Fabp7), while GCaMP6s signal was enhanced with an antibody against GFP. Scale bar = 50 µm. (**E**) Quantification of the percentage of GCaMP6s+ cells also positive for Fabp7. Data from *n* = 7 mice, four sections counted for each mouse. Standard deviation and mean values are shown in the graph. (**F**) Representative image of flow cytometry result of L3-L5 DRGs dissociated from the *Fabp7*-CreER-*Sun1GFP* line, with SGCs stained for GS. The events shown in the plot are single live cells positive for GFP. Forward side scatter height (FSC-H) on the *y*-axis and fluorescence from allophycocyanin (APC)–conjugated GS on the *x*-axis. See Supplementary Fig. 3A for full gating strategy. **(G)** Fluorescence-minus one control for **F**, showing the same sample without GS+ antibody stain. **(H)** Quantification of absolute numbers of Sun1GFP-expressing cells measured via flow cytometry of L3-L5 DRG, *n* = 5 mice. Standard deviation and mean values are shown in the graph. **(I)** Plot of the percentage of Sun1GFP+ cells also positive for GS+ (*n* = 5 mice). Error bars = standard deviation. **(J)** Quantification of flow cytometric results from dissociated DRGs from the *Fabp7*-CreER-*GCaMP6s* line, stained with markers for myeloid cells (CD11b+), endothelial cells (CD31+), immune cells (CD45+) and SGCs (GS+). Standard deviation and mean values are shown in the graph. See Supplementary Fig. 4 for full gating strategy. **(K)** Representative image of an immunofluorescent staining experiment designed to probe for off-target GCaMP6s signal in the nerve trunk: myelin protein zero (MPZ), GFP + GCaMP6s, DAPI+ nuclei and Fabp7+ SGCs. The encircled area is identified based on the high immunoreactivity for MPZ and is where the nerve enters the DRG. This region is rich in axons, fibroblasts and Schwann cells. Scale bar = 100 µm. **(L)** Quantification of the percentage of GCaMP6s+/Fabp7− cells found in the area stained by MPZ. The number of cells was determined by counting the number of nuclei. Four sections were counted per mouse, and the resulting percentages were plotted here from *n* = 7 mice. Standard deviation and mean values are shown in the graph.

First, we validated the *Fabp7*-CreER driver line to ensure that it is specific for SGCs. For this, we generated two crosses: *Fabp7*-CreER with flox-STOP-*GCaMP6s* for immunofluorescence and calcium-imaging experiments, and *Fabp7*-CreER with the nuclear-tagged reporter line flox-STOP-*Sun1GFP*. The latter reporter line is a superior tool for flow cytometry compared with the GCaMP6s reporter, since its GFP is constitutively active. We found that *Fabp7*-CreER is only a very weak driver of recombination, with only about half of all tamoxifen-treated mice showing the expected reporter gene expression. Mice with insufficient reporter gene labelling were excluded from data collection and/or analysis. Immunostaining of DRGs from successfully recombined *Fabp7*-CreER-*GCaMP6s* mice (*n* = 6–7) showed that an average of 76.5% of GCaMP6s-positive cells were also positive for the SGC marker GS (Fig. 1A–C) and that 85% of GCaMP6s-positive cells were positive for Fabp7 (Fig. 1D and E). For the GS staining, similar results were obtained via flow cytometry of *Fabp7*-CreER-*Sun1GFP* mice (*n* = 5), revealing 68.5% GS positivity among GFP+ cells (Fig. 1F–I and Supplementary Fig. 3A).

To test for off-target expression of the *Fabp7*-CreER driver, we performed further flow cytometry and immunofluorescence experiments. With flow cytometry, among the GFP+ population, 0.41% of cells were also positive for the myeloid cell marker CD11b, 1.1% for the endothelial cell marker CD31 and 0.5% for the pan-immune cell marker CD45 (Fig. 1J). Using this more extensive flow panel (Supplementary Fig. 4), we detected fewer GS+ cells among the Sun1-GFP+ population (44% on average). We suspect that this was due to spectral interference, which reduced our precision for distinguishing GS+ from GS− cells in this particular experimental run. Using immunostaining, we checked for GCaMP6s expression in the area where the nerves enter the DRG (Fig. 1K). On average, we found 0.07% of the cells in this area to be GCaMP6s + Fabp7− (*n* = 7; Fig. 1L), suggesting that there is little or no off-target expression in Schwann cells and fibroblasts. The specificity of the expression is in line with data previously presented by Avraham *et al*.^15^ and Mapps *et al*.^16^

**Figure 2:**
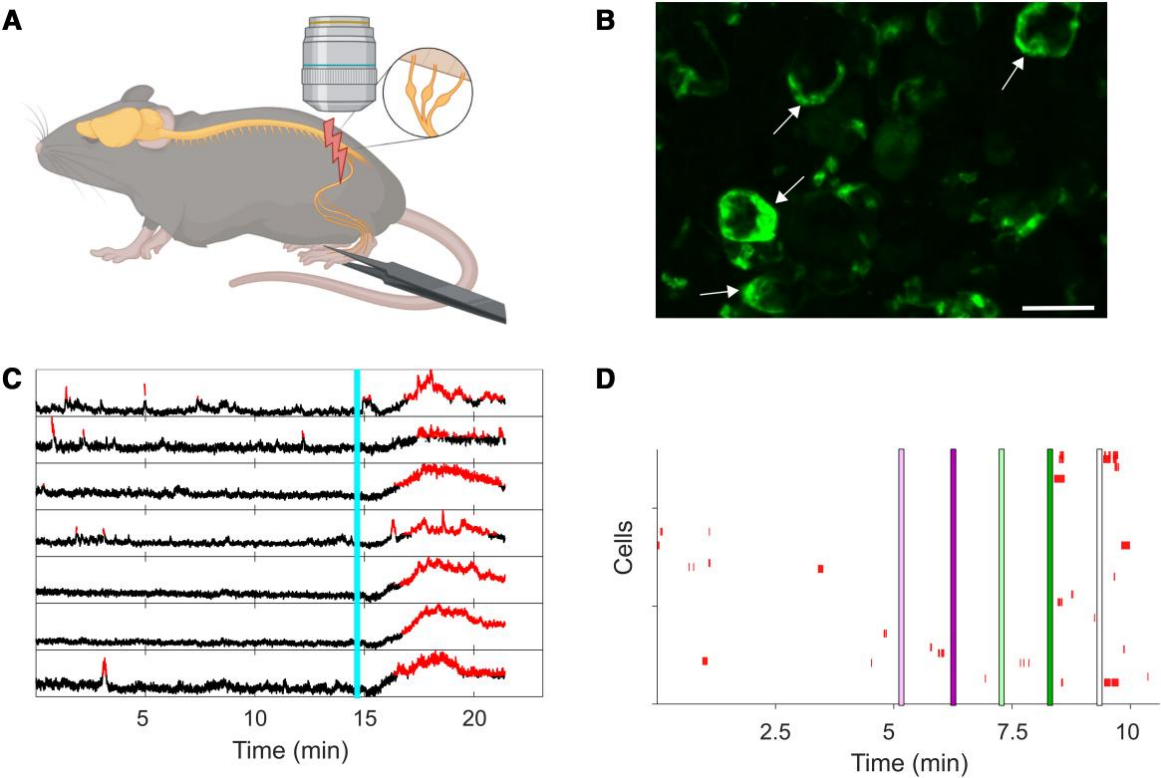
***In vivo* Ca^2+^ imaging of SGCs. (A)** Diagram of the experimental setup. *Fabp7*-CreER-*GCaMP6s* mice were anaesthetized, their L4 DRG exposed and imaged using an upright confocal/multiphoton microscope as described previously.^20^ The enlargement shows L3–L5 DRGs that contain the cell bodies of neurons within the sciatic nerve. Neurons were activated using electrical and natural stimuli at the level of the sciatic nerve. Created with BioRender.com. **(B)** Max projection image of exposed DRG at 20× magnification. Scale bar = 50 µm. The arrows point to clear examples of SGCs surrounding neuronal cell bodies. **(C)** Representative fluorescent traces from seven individual SGCs during an imaging session. The turquoise line indicates injection of an overdose of pentobarbital resulting in a post-mortem influx of calcium. The red parts of the traces show when the fluorescence signals are higher than the background indicating a response. The threshold was set as 25% above the mean + 2 × SD based on visual inspection of the recorded videos. **(D)** Representative raster plot of all SGCs recorded from one mouse (for this mouse *n* = 127 cells). Each red colour annotation represents a significant Ca^2+^ signal in an individual SGC. Coloured lines (pink, green or white) indicate the times at which neurons were stimulated: single A fibre stimulation (light pink line), 10 s of A fibre stimulation (5 Hz pulses, dark pink line), single suprathreshold fibre stimulation (light green line), 10 s of suprathreshold stimulation (5 Hz pulses, dark green line) and squeezing of the paw (5 s, white line).

Overall, these analyses indicated that GCaMP6s+/GS− cells are likely to represent GS-negative SGCs,^15^ rather than ectopic expression of GCaMP6s in other cell types. However, while we can thus assume with reasonable confidence that GCaMP6s+ cells are SGCs in *Fabp7*-CreER-*GCaMP6s* DRG, the same cannot be said for the reverse: in our hands, many SGCs in *Fabp7*-CreER mice were not positive for GCaMP6s and/or Sun1GFP. Specifically, in the flow cytometric data on average, only 7.8% of GS-positive SGCs were also positive for Sun1GFP (Supplementary Fig. 3B). For both lines, we found very sparse and variable labelling, likely due to the lack of the T2 mutations that were introduced into the CreER fusion protein by Pierre Chambon and Daniel Metzger in the late 1990s.^40,41^ Bearing these restrictions in mind, we proceeded to *in vivo* calcium-imaging experiments. Calcium imaging of lumbar L4 DRG was performed in live anaesthetized mice using an upright confocal/multiphoton microscope with a fully open pinhole, as described previously (Fig. 2A).^20^ In well-labelled mice (24 mice of 60), GCaMP6s+ cells could readily be identified by eye and had clear SGCs morphology (Fig. 2B). Calcium signal was readily discernible against the background in most cells (Fig. 2C and Videos 1–3), with only 13 of 1418 SGCs displaying baseline activity high enough to prevent the use of our set response threshold. The threshold was determined based on visual inspection of our videos to ensure that only true fluorescence signals would be included in subsequent analyses. It was set as the signal at 25% + 2 × Standard derivation above the mean cell fluorescence over the entire recording period or until injection of pentobarbital. Plotting responses across *n* = 6 naïve animals (two males, four females), we could not detect any discernible connection between SGC calcium signal and electrical stimulation of neurons at A and C fibre frequencies (Fig. 2D and Supplementary Fig. 1). Instead, it seemed that SGCs responded at arbitrary intervals throughout the recording period regardless of neuronal stimulation.

This changed somewhat once peripheral neurons were sensitized, either through injection of an inflammatory stimulus into the paw (CFA, 20 μL) or after traumatic nerve injury as a result of pSNL. One day after CFA or 7 days after pSNL, we observed a higher number of time-locked calcium responses than expected by chance, but only after repeated suprathreshold stimulation (Table 4). A time-locked response was defined as a Ca^2+^ response initiated within 7.5 s of the noted time for the stimulation of the neurons. There was not a higher number of time-locked responses than expected for A fibre, single suprathreshold or paw pinch stimulation.

**Table 4:** The likelihood of observing a time-locked response by chance (‘Exp. by chance’) and the actual observed responses (‘Obs. in data’) in the collected data

	Chance per cell	Number of cells	Exp. by chance in data	Obs. in data: A fibre	Obs. in data: A fibre repeated	Obs. in data: suprathreshold	Obs. in data: suprathreshold repeated	Obs. in data: paw squeeze
Na**ï**ve	0.011	368	4	2	2	2	3	3
CFA	0.0075	533	4	2	2	2	11	5
pSNL	0.014	517	7	4	5	4	9	5

‘Chance per cell’ shows the theoretical chance that one cell appears time-locked based on the frequency of the responses and the length of the recording. ‘Number of detected cells’ states the number of cells recorded for each condition in all videos. ‘Exp. by chance in data’ is the number of times a cell will appear time-locked by chance. ‘Obs. in data’ is the number of times a time-locked response was recorded in the data after electrical stimulation or paw squeeze. Obs., observed; Exp, expected.

**Figure 3:**
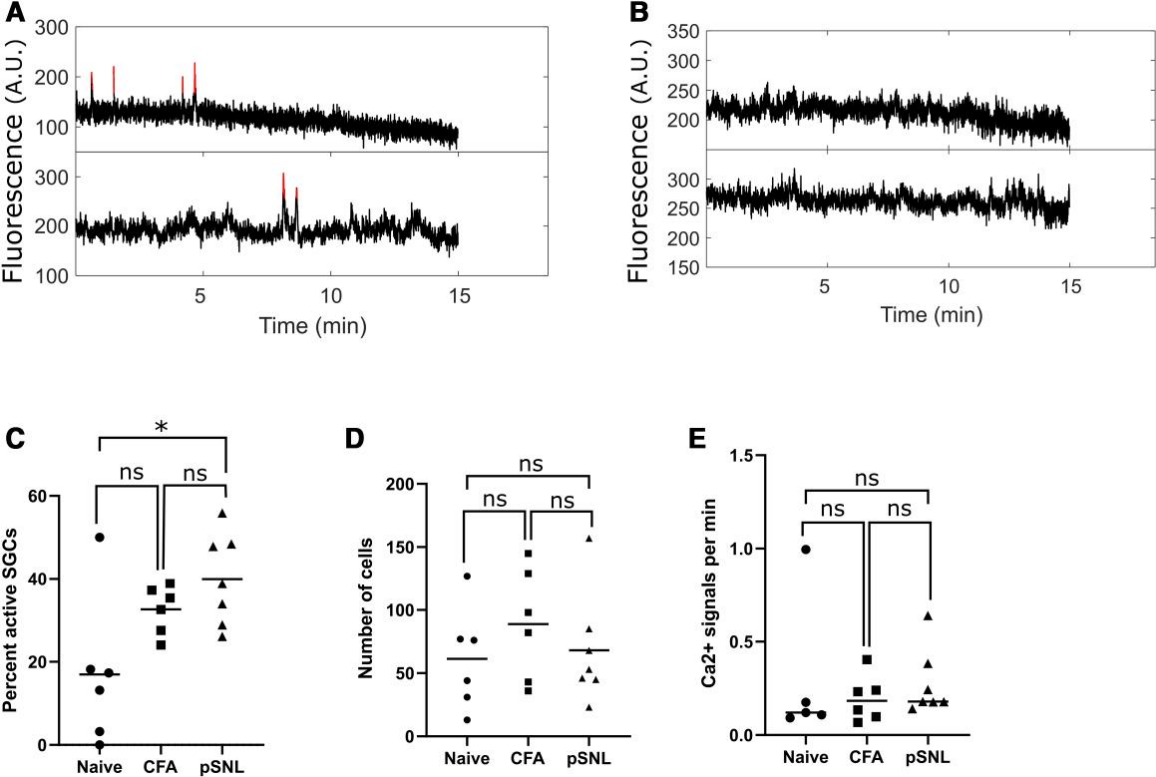
**Quantification of Ca^2+^ responses in SGCs after peripheral sensitization. (A)** Two example traces of active SGCs in the baseline condition, i.e. without stimulation. The red lines indicate responses above background noise. Fluorescence is shown in arbitrary units (AUs). **(B)** Two example traces of inactive SGCs. **(C)** Percent SGCs that have at least one Ca^2+^ response in each mouse across the conditions: naïve (*n* = 6 mice), CFA (*n* = 6 mice) and pSNL (*n* = 7 mice). Ordinary one-way ANOVA test followed by Tukey’s multiple comparisons test of means comparing naïve versus CFA (*P* = 0.114), naïve versus pSNL (*P = 0.01) and CFA versus pSNL (*P* = 0.55). **(D)** The number of SGCs imaged in each mouse across the three conditions. Ordinary one-way ANOVA test followed by Tukey’s multiple comparisons test of means comparing naïve versus CFA (*P* = 0.52), naïve versus pSNL (*P* = 0.96) and CFA versus pSNL (*P* = 0.67). **(E)** The average number of Ca^2+^ signals per minute across active SGCs within each mouse. SGCs with at least one Ca^2+^ response were considered active and thus included in this plot. Ordinary one-way ANOVA test followed by Tukey’s multiple comparisons test of means comparing naïve versus CFA (*P* = 0.77), naïve versus pSNL (*P* = 0.99) and CFA versus pSNL (*P* = 0.81).

Next, we looked across the entire recorded period to determine whether each SGC was, at any timepoint, responding with a calcium signal (Fig 3A and B). The pain models induced an increase in the percentage of responding SGCs (Fig. 3C), with the mean number of responders rising from 17% in naïve mice (*n* = 6) to 32.6% in CFA mice (*d* = 1.25, *n* = 6) and 40% in pSNL mice (*d* = 1.84, *n* = 7). This change was statistically significant for the pSNL versus naïve comparison using an ordinary one-way ANOVA test followed by Tukey’s multiple comparisons test of means (*P* = 0.01). This change was not due to a change in the total number of SGCs that were included in the analysis of any given group (Fig. 3D). Moreover, sensitization did not appear to increase the frequency of responses in any individual SGC (Fig. 3E).

**Figure 4:**
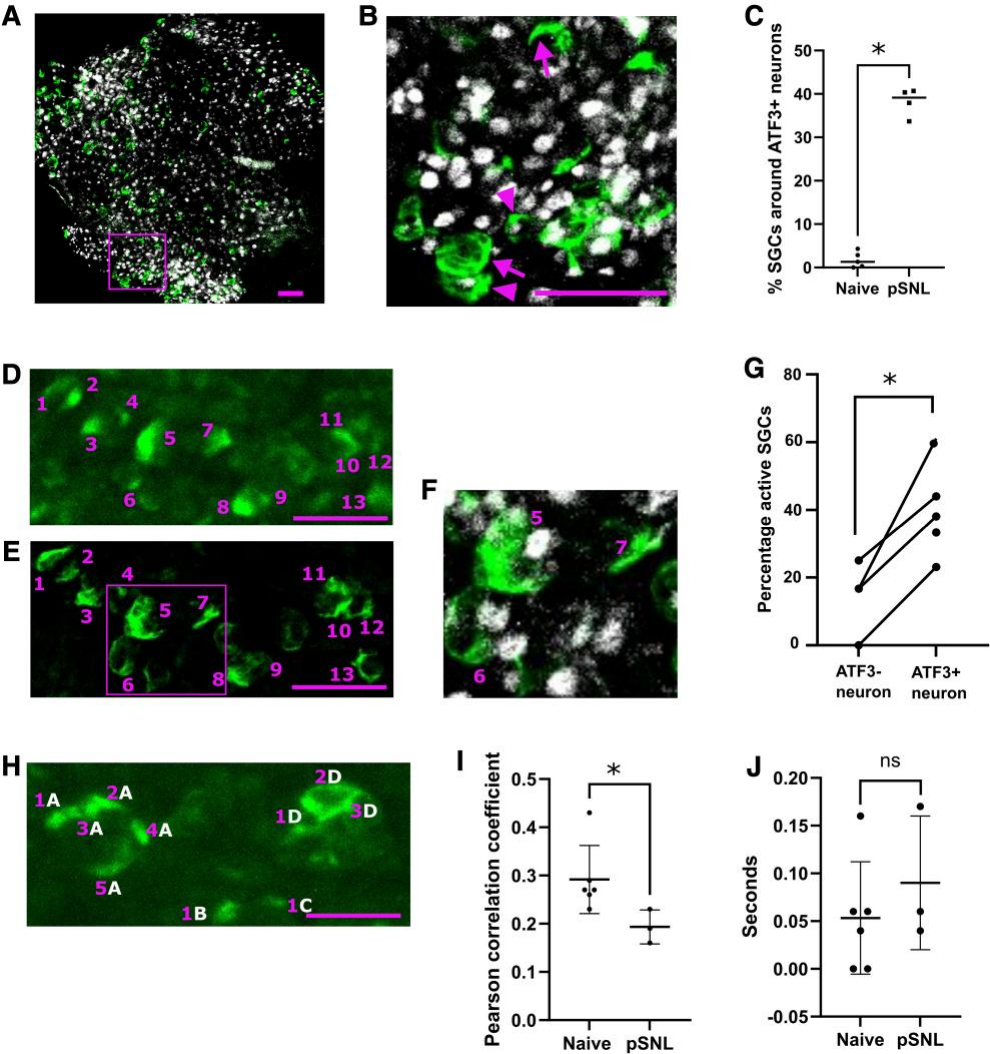
**Analysis of SGCs surrounding injured or uninjured neurons after nerve ligation. (A**) Representative image of immunostained L4 DRG, following *in vivo* Ca^2+^ imaging. The tissue was post-fixed by immersion and stained with the injury marker activating transcription factor 3 (ATF3) in grey and GFP to enhance GCaMP6s signal in green. Scale bar = 100 µm. **(B)** Magnified view of the region highlighted within the purple box in A. SGCs surrounding uninjured (ATF3−) versus injured (ATF3+) neurons are highlighted by arrows versus arrowheads, respectively. Scale bar = 100 µm. **(C)** Quantification of percentages of SGCs surrounding ATF3+ neurons in naïve (*n* = 5) and pSNL conditions (*n* = 4). Each data point shows the quantification from one immunostained L4 DRG from one mouse after *in vivo* Ca^2+^ imaging. Mann–Whitney test, *P = 0.016. **(D)** Representative image of SGCs during *in vivo* Ca^2+^-imaging recording with SGCs shown in green. The recorded SGCs are labelled with purple numbers. Scale bar = 100 µm. **(E)** Confocal image of whole-mount DRG stained against GFP to enhance GCaMP6s signal (green) in SGCs following imaging. SGCs were matched to their *in vivo* Ca^2+^-imaging profile, i.e. purple cells 1–13 imaged via confocal in E are the same as purple cells 1–13 in D, recorded during an imaging session. Scale bar = 100 µm. **(F)** Magnified view of the region highlighted with purple in E. The magnification highlights SGC numbers 5–7 including the immunostaining of the injury marker ATF3 (grey) to identify which SGCs surround injured neurons. SGC 5 surrounds an ATF3+ neuron, SGC 6 is undetermined and SGC 7 surrounds an ATF3− neuron. **(G)** The percentage of active SGCs surrounding either ATF3+ or ATF3− neurons after nerve injury. Data derived from *n* = 4 videos from three mice; paired *t*-test; *P = 0.019. Each set of paired data points shows the quantified percentage of active SGCs around either ATF3+ or ATF3− neurons in each video. In the fourth pSNL mouse plotted in C, no SGCs surrounding ATF3− neurons were identified in the corresponding video. It could therefore not be included in the analysis. **(H)** Representative image of SGCs during *in vivo* Ca^2+^ recording with SGCs shown in green. The SGC-neuron units are marked with white letters, and individual SGCs in each unit are marked with purple numbers. The individual SGCs are identified with Suite2p based on their Ca^2+^ activity. Scale bar = 100 µm. **(I)** The recorded Ca^2+^ fluorescent traces from SGCs in the same unit were compared with Pearson’s correlation. The graph shows the mean correlation between SGCs in the same unit in each animal. The *y*-axis shows the correlation coefficient *R*. Data were derived from *n* = 6 mice from the naïve group and *n* = 3 mice from the pSNL group. In the pSNL group, SGCs were only considered if they surrounded an injured neuron. Each data point represents the mean correlation in each mouse from between 4 and 103 correlations. Statistical analysis was performed using a non-parametric, two-tailed Mann–Whitney test, *P = 0.048. **(J)** The graph shows the mean lag time with the highest correlation between two SGCs surrounding the same unit in each animal. The *y*-axis shows the lag time in seconds. Data were derived from the same cells as in Fig. 1. Statistical analysis was performed using non-parametric, two tailed Mann–Whitney test, *P* = 0.46.

After *in vivo* imaging, DRGs from the pSNL animals were used to quantify how many of the SGCs were in fact surrounding injured neurons. To perform this analysis, DRGs were immersion fixed in 4% PFA and stained with an antibody against the injury marker ATF3 (Fig 4A and B). We observed that slightly less than half of the SGCs that we recorded in pSNL animals surrounded injured neurons (mean = 38%), while the remaining 62% surrounded uninjured neurons (Fig. 4C). This means that the size of the effects plotted in Fig. 3C may be an underestimate, since our data include many SGCs that were not in the proximity of sensitized neurons.

To address this, we used careful alignment to overlap the *in vivo* calcium signals we observed with the ATF3 labelling post-mortem. By combining these two imaging modalities, we were able to compare the activity of SGCs surrounding uninjured versus injured neurons in the same DRG (Fig. 4D–F). We found that the percent of active SGCs surrounding injured neurons was larger than that surrounding uninjured neurons (*d* = 2, *n* = 4; Videos 1–3 and Fig. 4G). As expected from this within-animal comparison, the effect was slightly larger than that observed in our less sensitive, between-animal comparison (Fig. 3C).

To investigate whether we could detect a rise in intracellular calcium waves exchanged between SGCs surrounding the same injured neuron, we assessed whether they are more correlated in their activity compared with SGCs that surround the same naïve neuron. First, we identified SGCs surrounding the same neuron (Fig. 4H) and whether they were surrounding an injured or uninjured neuron based on neuronal ATF3 expression. Next, we calculated the Pearson correlation of the calcium traces from SGCs surrounding the same neuron. The resulting data did not provide any evidence for a rise in intracellular calcium waves in the injured animals; on the contrary, the mean correlation in activity was higher for SGCs surrounding naive rather than injured neurons (Fig. 4I). To understand if the low correlation in the injured animals was due to a time delay in the spread of Ca^2+^ waves as reported^7^ and shown in astrocytes,^42^ we performed a series of correlations for each pair of SGCs surrounding the same neuron. For each of these cross-correlations, we shifted the recordings one frame at a time, to capture a variety of possible time lags—up to a maximum of 2 s. We then plotted the highest correlation for each SGC pair and found no systematic differences could be discerned in terms of time lag (Fig. 4J). That is, for the most part, the activity in two SGCs surrounding the same cell was best correlated with a mean time lag of 0.07 s, with no significant differences between experimental groups (naïve versus pSNL).

## Discussion

Our study set out to use a transgenic mouse model to image SGC calcium transients *in vivo*. Our results are the first to show in a whole-animal physiological setting that there is more SGC activity in a sensitized peripheral nervous system. Specifically, an increased percentage of active SGCs could be observed in inflammation (CFA) and nerve injury (pSNL) models compared with naïve controls. Moreover, a higher percentage of SGC-generated calcium transients could be observed around ATF3+ injured neurons, compared with their uninjured, ATF3− counterparts. However, with the exception of repeated suprathreshold stimuli, the response of individual SGCs did not appear to be time-locked with actual neuronal firing.

The general lack of time-locked SGC calcium transients suggests that they are not directly induced by neuronal action potentials. This finding is surprising, since it conflicts with prior *in vitro* work.^21-23^ For example, it has been shown by Zhang *et al*.^21^ that cultured DRG neurons release ATP from their soma upon firing, while the SGCs in turn detect the ATP and respond with an intracellular calcium signal. One reason for this discrepancy could be that the neurons in the culture are electrically activated for 30 s, while we did electrical pulses for no longer than 10 s. This explanation is also supported by the observation that we do see some time-locked responses in the injured condition with repeated suprathreshold stimuli, which is an extraordinary physiological situation.

The *in vivo* system we used in this study has some challenges. Specifically, the transgenic mouse model has sparse labelling of SGCs, and the neuronal cell bodies in the imaged DRG do not all project to the sciatic nerve.^43^ In theory, these two challenges could mean that we have missed time-locked signals. However, we think this is unlikely, since we were able to activate ∼75% of labelled neurons with our electrical stimulation paradigm (437 neurons of 575 labelled neurons from 2 mice; Supplementary Fig. 1) and have imaged over 1378 SGCs from a total of 19 mice. Currently, we therefore cautiously conclude that neuronal-SGC signalling with Ca^2+^ as a second messenger may be less important in homeostatic conditions than we would have previously thought.

During peripheral sensitization, we observe an overall, diffuse increase in SGC Ca^2+^ activity. The function of this is currently unknown. In general, intracellular Ca^2+^ concentrations are used as second messengers and can influence a broad variety of signalling pathways. We speculate that in this case, the increase in Ca^2+^ activity could be a reflection of the need for more buffering of the extracellular space in the SGC-neuron unit. This might be necessary to accommodate any extra ions and neurotransmitters released into the SGC-neuron unit, as a result of the increased neuronal activity known to occur after inflammation or nerve injury.^44^ In line with this, it has recently been suggested by Schulte *et al*.^45^ that SGCs undergo changes in metabolic function after nerve injury, evidenced by a change in marker expression of GFAP and GS.

Like our team, Chen *et al*.^46^ were also able to analyse calcium signals in SGCs. They did not analyse them at a single-cell level but examined aggregated calcium signals across a wider area, reporting an increase when ATP was applied directly to the DRG. While Chen *et al*. did not investigate pain models, their results raise the possibility that the increase in activity we found after inflammation or nerve injury might be linked to ATP released by injured neurons.

It should be noted that our model has some limitations. First, we focused our attention on calcium responses in SGCs, but there are many other ways in which these cells might functionally respond to neuronal activity, such as the release of messenger molecules. Secondly, our setup does not permit a distinction between increased SGC–SGC communication (e.g. via gap junctions) versus increased communication between one neuron and each of its SGCs individually. Thirdly, our imaging was done with mice under anaesthesia, which might dampen their responses, as reported in astrocytes.^47^ Finally, labelling of SGCs using the *Fabp7*-CreER mouse line proved extremely sparse and variable. This meant that the results described above were very time consuming to obtain, with every other animal labelled too poorly to proceed to imaging. Moreover, even in the animals that we could image, many SGCs would have remained unlabelled, which means that we would have missed other smaller effects in addition to the large changes we observed. On the other hand, the sparse labelling was an advantage in the challenging task of identifying individual SGCs. The close physical connection between SGCs surrounding the same neuron makes it difficult to discern individual SGCs when they are all labelled. For this task specifically, it was therefore helpful that *Fabp7*-CreER failed to drive gene expression in all SGCs. We also relied on the Ca^2+^ activity of the cells to distinguish them. This strategy is somewhat complicated by the fact that Ca^2+^ waves have been reported to spread from SGC to SGC via gap–junction coupling. While the waves have been observed to be separated in time,^7^ it is still conceivable that occasionally two separate SGCs would have been erroneously identified as one single cell.

## Conclusion

*In vivo* calcium imaging of SGCs has permitted us to visualize some of the functions of these cells in intact but anaesthetized mice, during health and disease. It appears that they work according to their own schedule but crucially increase their overall output in more metabolically demanding settings like inflammation and nerve injury. At present, it is not clear whether this increase in activity is maladaptive or not. Currently, not much is known about the downstream effects of Ca^2+^ signalling in SGCs, but their altered function in disease states is generally believed to enhance neuronal excitability and pain.^48^ The additional *in vivo* data we provide here thus strengthen the case for targeting SGC function pharmacologically, in order to bolster neuronal function and alleviate pain in neuropathy.

## Supplementary Material

fcae013_Supplementary_Data

## Data Availability

Raw fluorescence traces from Suite2p and MATLAB scripts are made available here: https://osf.io/jg5zr/? view_only.
