# Supplementary material for: In vivo calcium imaging shows that satellite glial cells have increased activity in painful states
Source: Brain Commun. 2024 Jan 18;6(2):fcae013. doi: 10.1093/braincomms/fcae013 (PMC11024818; doi:10.1093/braincomms/fcae013)
Supplement: fcae013_Supplementary_Data [file fcae013_supplementary_data.pdf]

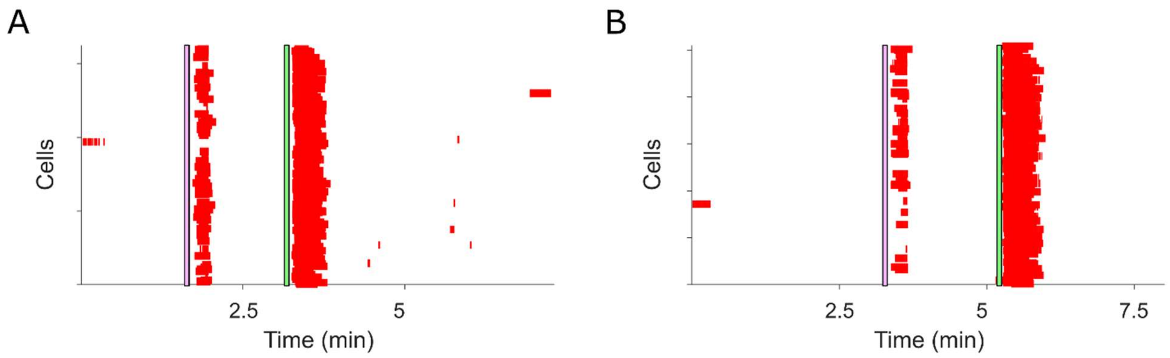

Supplementary Figure 1: In vivo Ca^{2+} imaging of neurons

(A)-(B) Raster plots showing neuronal activation following electrical stimulation. Each raster plot represents data from one mouse. The red annotations indicate significant Ca^{2+} signal above baseline. Coloured lines (pink and green) indicate times at which neurons were stimulated: A fibre stimulation for 10sec at 4 Hz (light pink), suprathreshold stimulation for 10sec at 4 Hz (light green).

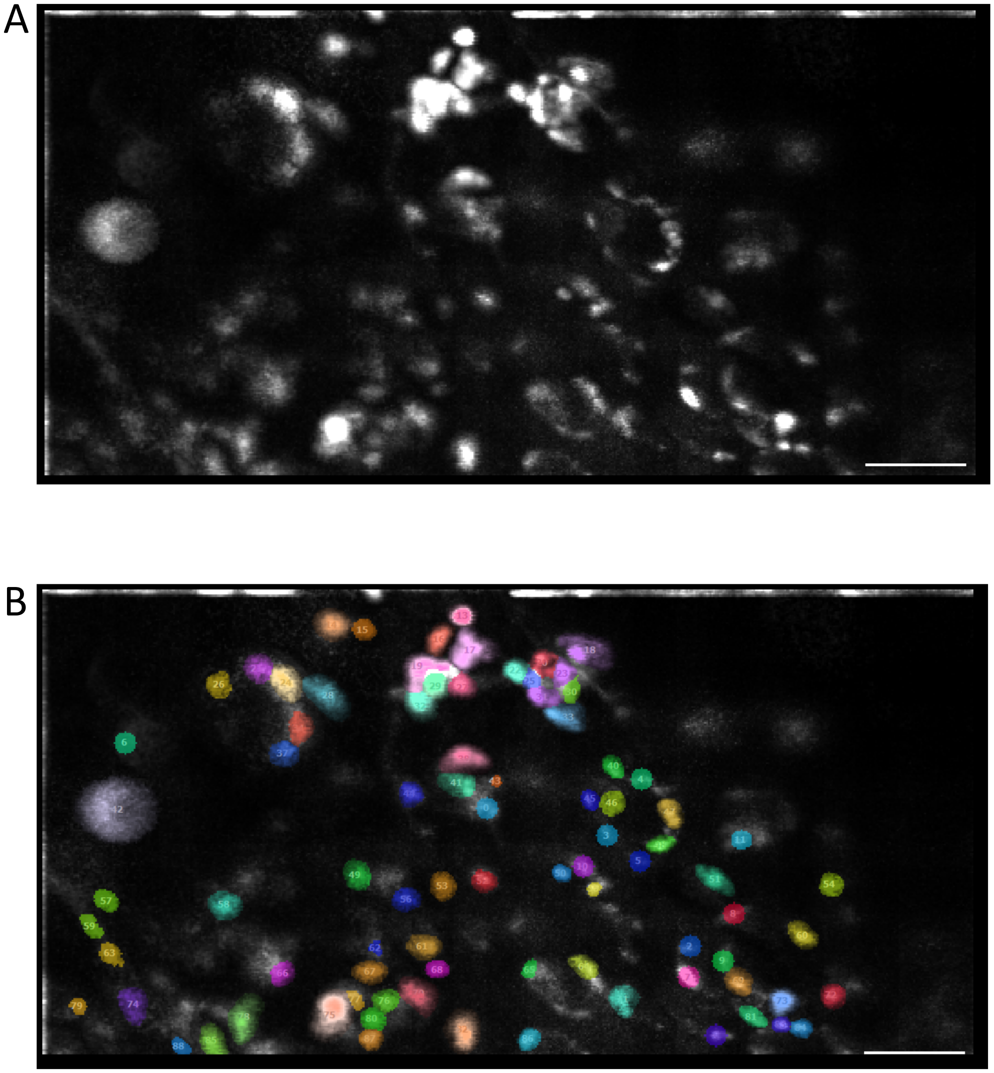

Supplementary Figure 2: Identification of individual SGCs with Suite2p and Cellpose

(A) Example of max projection of GCaMP6s signal in SGCs from *in vivo* Ca^{2+} imaging experiment. Scale bar = 100µm **(B)** Detected regions of interest (ROI) and ROI number overlaid on the max projection. Suite2p and Cellpose take the activity throughout the video into account when annotating the ROIs, which ensures that individual SGCs can be distinguished when they fill with calcium at different times, either throughout the recording or as the mouse is culled at the end. Scale bar = 100µm.

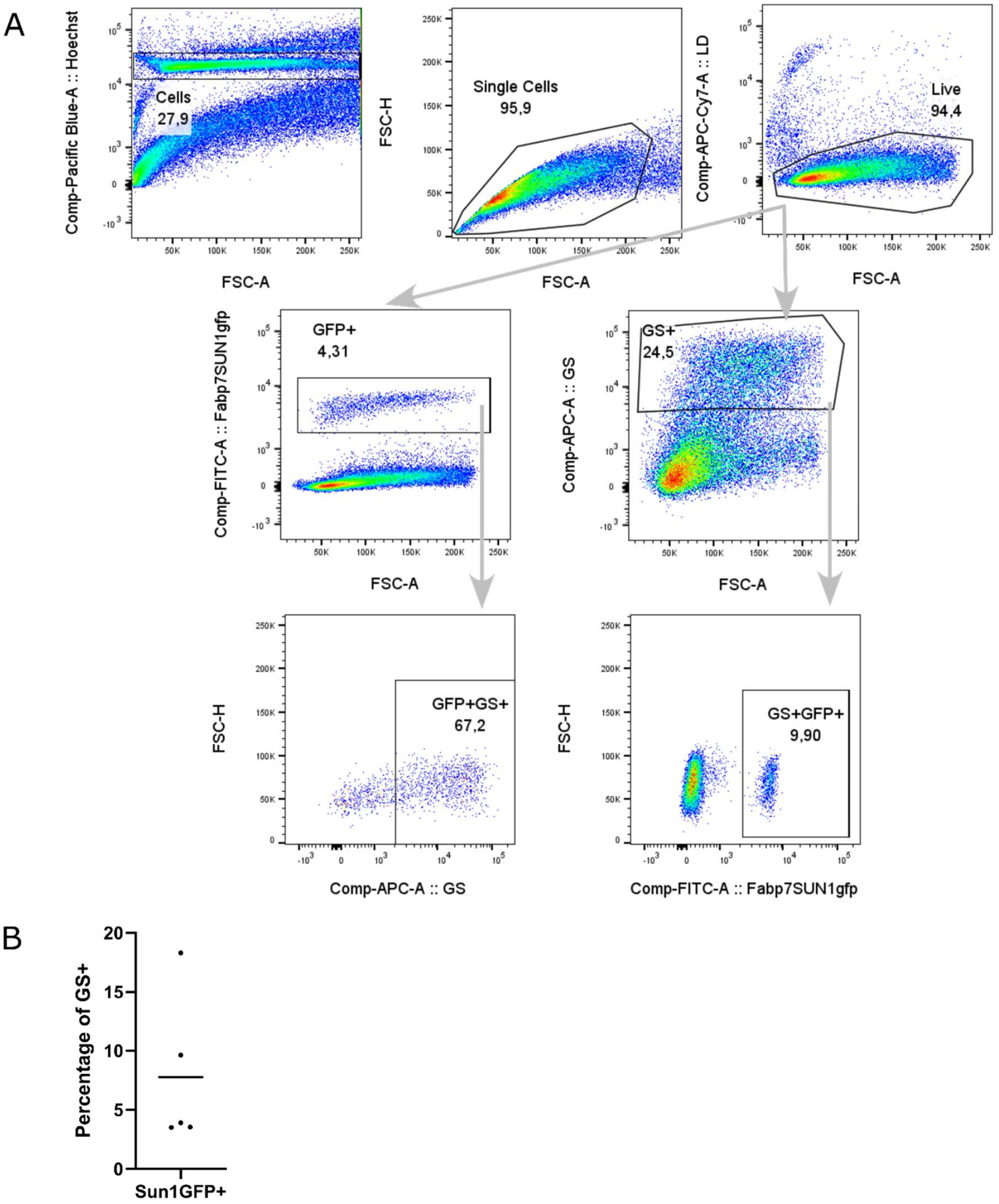

Supplementary Figure 3: Gating strategy for flow cytometric experiment (small antibody panel)
(A) Gating strategy for Figure 1D. Samples run on a BD FACSCanto. First, cells are distinguished from debris with Hoechst staining. Next, single cells followed by live cells are identified. From here either the *Fabp7*-CreER-*Sun1GFP* positive cells or the GS positive cells are identified and lastly the percentage of Sun1GFP and GS double positive cells are determined. All gates are placed based on fluorescence-minus-one (FMO) controls. **(B)** Quantification of the percentage of single, live and GS positive cells that are also positive for Sun1GFP. The black line represents the mean percentage, *n*=5 mice.

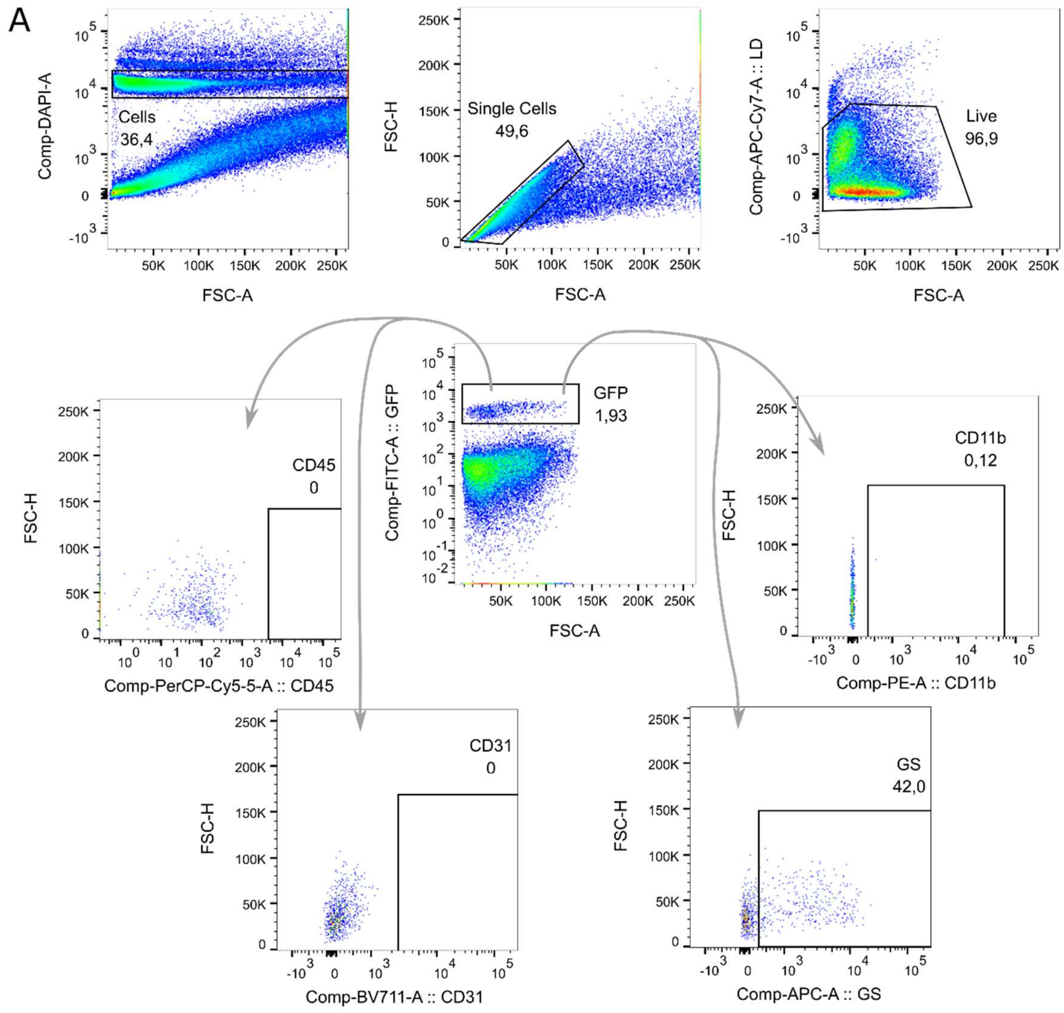

Supplementary Figure 4: Gating strategy for flow cytometric experiment (big antibody panel)
(A) Gating strategy for Figure 1H. Samples run on a BD Fortessa. In the first three plots Hoechst positive, singlets and live cells are identified. Next, *Fabp7*-CreER-*Sun1GFP* positive cells are identified. From there, the percentage of GFP+ cells also positive for the cell markers CD11b, CD45, CD31 and GS are determined. All gates are placed based on FMO controls.
